# Formulation of PVA Hydrogel Patch as a Drug Delivery System of Albumin Nanoparticles Loaded with Curcumin

**DOI:** 10.3390/gels11120979

**Published:** 2025-12-05

**Authors:** Lyubomira Radeva, Aleksandar Belchev, Parsa Karimi Dardashti, Yordan Yordanov, Ivanka Spassova, Daniela Kovacheva, Mariya Spasova, Petar D. Petrov, Virginia Tzankova, Krassimira Yoncheva

**Affiliations:** 1 Faculty of Pharmacy, Medical University of Sofia, 1000 Sofia, Bulgaria; ale.belchev@gmail.com (A.B.); pk.dardashti@yahoo.com (P.K.D.); yyordanov@pharmfac.mu-sofia.bg (Y.Y.); vtzankova@pharmfac.mu-sofia.bg (V.T.); 2 Institute of General and Inorganic Chemistry, Bulgarian Academy of Sciences, 1113 Sofia, Bulgaria; ispasova@svr.igic.bas.bg (I.S.); didka@svr.igic.bas.bg (D.K.); 3 Institute of Polymers, Bulgarian Academy of Sciences, 1113 Sofia, Bulgaria; mspasova@polymer.bas.bg (M.S.); ppetrov@polymer.bas.bg (P.D.P.)

**Keywords:** curcumin, albumin, nanoparticles, PVA, patch, skin application

## Abstract

Curcumin is a widely researched natural molecule due to its abundance of pharmacological effects, such as antioxidant, antitumor, anti-inflammatory, etc. The main limitation of curcumin, however, is its low aqueous solubility, which worsens its biopharmaceutical characteristics. The aim of this study was to encapsulate curcumin in albumin nanoparticles and to subsequently incorporate them into a polyvinyl alcohol patch, resulting in a new drug formulation for skin application. The nanoparticles were characterized by a small mean diameter of approximately 162 nm, a narrow size distribution, and a negative zeta potential. TEM confirmed the small size of the nanoparticles. The ratio between the drug and albumin was optimized, achieving approximately 88% encapsulation efficiency. Protein–ligand docking, utilizing CB-Dock, indicated a strong interaction between curcumin and albumin. The binding between the molecules was proved via diffuse-reflectance UV–vis and XRD analyses. The encapsulated curcumin showed a significantly potentiated scavenging activity against ABTS and DPPH radicals in comparison with the pure drug, as well as a protective effect in H_2_O_2_-induced oxidative stress in fibroblasts. The loaded nanoparticles were further incorporated in a PVA hydrogel patch, which was characterized in terms of mechanical properties and in vitro release. Therefore, the resulting system could provide more effective skin delivery and an improved antioxidant activity of curcumin.

## 1. Introduction

The effective treatment of skin disorders is a vastly important issue due to their negative impact on patients’ quality of life. Skin is the largest human organ and plays an important protective role from external environmental factors. Therefore, it is significantly susceptible to different negative influences, such as ultraviolet rays, oxygen, pollution, as well as xenobiotics. Oxidative stress considerably contributes to the pathogenesis of skin diseases [1,2,3]. Thus, the application of natural antioxidants for the prevention and treatment of skin disorders could be considered a suitable strategy. Natural antioxidants possess a significant scavenging activity against reactive oxygen species (ROS) and, at the same time, show a good toxicological profile [4,5,6]. However, many of these substances exhibit serious biopharmaceutical challenges, including poor aqueous solubility, low stability, and compromised pharmacological effects. Thus, novel technological approaches could be implemented to address these issues.

Curcumin is a typical natural antioxidant, in particular a polyphenol derived from *Curcuma longa* (turmeric). Its mechanism of action is related to the scavenging of ROS and reactive nitrogen species (RNS), inhibition of lipid peroxidation, chelation of metal ions, and activation of genes that code antioxidant enzymes, such as superoxide dismutase, catalase, glutathione peroxidase, and glutathione-S-transferase [7,8]. Its potential in dermatology for the treatment of psoriasis, dermatitis, infections, acne, skin ageing, wound healing, and skin cancer has been reported [9,10]. However, despite its antioxidant potential and good toxicological profile, curcumin possesses limited aqueous solubility that hinders its application in pharmaceutical technology [11]. The incorporation of curcumin in nanoparticles is an established approach to increasing its solubility and enhancing its application [12]. Polymeric nanoparticles, particularly those prepared from natural polymers, are considered appropriate for skin application because they can protect the drug from premature degradation, control permeation through the stratum corneum, provide modified release, and enable passive or active targeted delivery [13]. The advantages of this type of nanoparticles compared to other types of topical systems, e.g., lipid-based nanoparticles or nanoemulsions, are related to higher stability in physiological conditions, more opportunities for sustained release, and reduced irritation [14,15]. Thus, various types of polymeric nanoparticles were investigated as carriers for the topical delivery of curcumin. For instance, curcumin was loaded in chitosan nanoparticles intended for tissue regeneration in burn wounds [16], zein-silk sericin nanoparticles for the treatment of atopic dermatitis [17], and poly(ε-caprolactone)-b-poly(ethylene glycol)-b-poly(ε-caprolactone) (PCL-PEG-PCL) nanoparticles for wound healing [18,19]. Albumin is an example of a natural polymer, in particular a globular protein, which is widely used as a carrier in pharmaceutical nanotechnology. It is biodegradable, non-toxic, and approved by the FDA for clinical use [20]. Moreover, the presence of functional groups in its structure provides an opportunity for interactions with the loaded drugs, which could result in high encapsulation efficiency and sustained release [21]. For example, bovine serum albumin (BSA) nanoparticles were loaded with amphotericin B for the treatment of cutaneous leishmaniosis with reduced toxicity [22] or with curcumin for the treatment of melanoma [23].

Another important aspect of effective skin disease treatment is the optimal application of the therapeutic agent on the skin. Hydrogel patches are considered appropriate for such an application due to the opportunity for homogenous adhesion to damaged skin and easy removal without affecting the regenerated skin [24]. For example, sodium carboxymethylcellulose and hydroxypropyl methylcellulose hydrogel patches were designed for the delivery of methotrexate for psoriasis treatment [25], polyvinyl alcohol (PVA)/collagen composite films were loaded with curcumin-PCL-PEG-PCL nanoparticles [19], PVA/chitosan patches were loaded with free curcumin for wound healing [26], and gelatin hydrogel film was loaded with bovine serum albumin/silver nanoparticles for the photothermal treatment of skin cancer [27]. Polyvinyl alcohol is a widely used polymer due to its aqueous solubility, biocompatibility, adhesiveness, and good mechanical properties [19,26,28]. Therefore, it is considered a very appropriate polymer for the preparation of hydrogel patches.

Taking all of this into consideration, the current research presents the encapsulation of curcumin in albumin nanoparticles and their subsequent incorporation in a PVA patch for skin application. The ratio between the drug and the biopolymer was optimized, resulting in high encapsulation efficiency. The system was characterized via DLS, TEM, and in vitro release analyses. Protein–ligand docking suggested a strong interaction between curcumin and albumin, confirmed by a sustained release and diffuse-reflectance UV–vis analysis. The successful encapsulation was verified via XRD analysis. The antioxidant capacity of the loaded curcumin was evaluated via the ABTS and DPPH assays, as well as in an in vitro cell model of oxidative stress. The PVA patches were characterized regarding their mechanical properties and in vitro release.

## 2. Results and Discussion

### 2.1. Preparation and Characterization of the Nanoparticles

**Figure 1:**
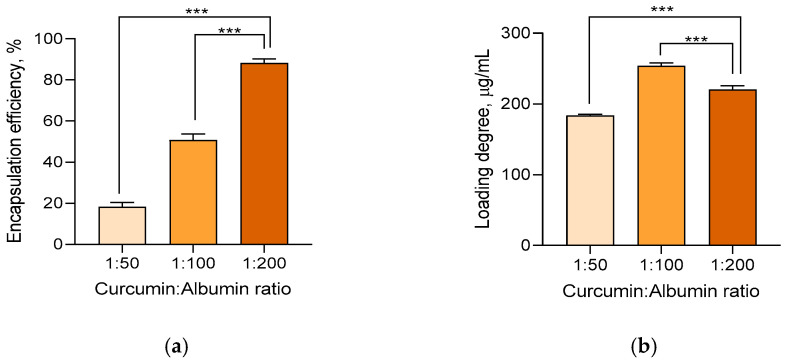
Encapsulation efficiency (**a**) and drug loading (**b**) of curcumin in albumin nanoparticles at different ratios. *** *p* < 0.001 between the groups.

The preparation of curcumin–albumin nanoparticles (CurcAlbNPs) was conducted via the desolvation method by applying acetone as an anti-solvent. In order to evaluate the impact of the drug:polymer ratio (wt/wt) on the efficacy of the encapsulation process, three ratios between curcumin and albumin, namely 1:50, 1:100, and 1:200, were examined. As shown in Figure 1a, a linear proportional relationship was observed by increasing the polymer part of the ratio. At the 1:200 ratio, the highest encapsulation efficiency of 88.2% was achieved. Regarding the loading degree, the highest value was observed for the 1:100 ratio (254.1 µg/mL), followed by 1:200 (220.5 µg/mL) and 1:50 (183.5 µg/mL) ratios (Figure 1b). Thus, the nanoparticles obtained at the 1:200 ratio were used for the following experiments because of the lower loss of the active substance. A similar relationship between drug:polymer ratio and loading efficiency was observed for rifampicin loaded in poly(lactic-co-glycolic-acid) (PLGA) nanoparticles [29], and coumarin 6 loaded in poly(caprolactone)-b-poly(ethylene oxide) (PCL-b-PEO)- or poly(styrene)-b-poly(ethylene oxide) (PS-b-PEO)-based nanoparticles [30].

**Figure 2:**
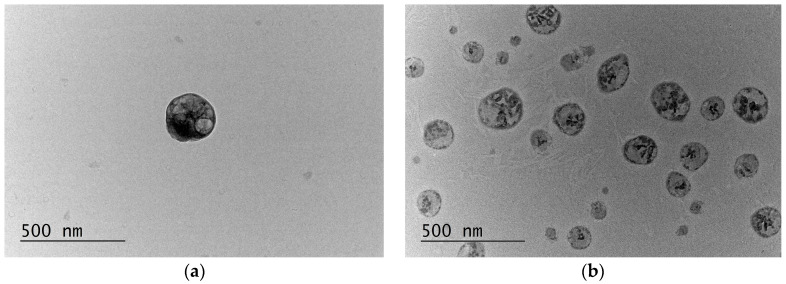
TEM images of empty (**a**) and curcumin-loaded (**b**) albumin nanoparticles.

The main characteristics of the nanoparticles are shown in Table 1. Both the empty and loaded with curcumin nanoparticles were characterized by a mean diameter below 200 nm, namely approximately 117 and 163 nm, respectively. An increase in the size was observed after the loading, which was probably due to the interactions between curcumin and albumin, leading to changes in the structure morphology. Both systems possessed a narrow size distribution with a lower polydispersity index for CurcAlbNPs. The zeta potential was negative, in particular −27.5 mV for the empty and −28.5 mV for the loaded albumin particles. The nanoparticles were also evaluated via transmission electron microscopy (TEM), which confirmed their small diameters and demonstrated an almost spherical shape of the particles (Figure 2).

### 2.2. In Vitro Release Study of CurcAlbNPs

**Figure 3:**
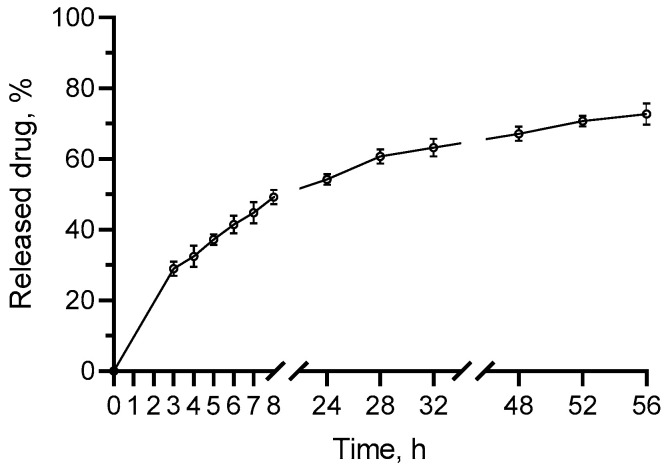
In vitro release profile of curcumin from albumin nanoparticles in medium with pH = 5.

The in vitro release test revealed sustained release for more than 50 h without a burst effect (Figure 3). In particular, there was approximately 29% released curcumin after 3 h and 73% after 56 h. Similar sustained release from albumin nanoparticles was observed for hydrophobic substances such as the flavonoid scutellarin (25 h) [31], and the curcumin derivative 2,6-divanillylidenecyclohexanone (165 h in pH = 5.5) [32]. A possible reason for this observation is the hydrophobic inner core of albumin nanoparticles, which provides opportunities for strong interactions with the hydrophobic curcumin [33]. It was discovered that during the formation of albumin nanoparticles, highly hydrophobic domains are formed via aggregation or nucleation, which is proven by the decreased exposure of the amino acid tryptophan residues to a solvent [33]. Moreover, this study discovered a coupling between tryptophan and the model hydrophobic substance dimethylcurcumin, which confirms the loading in the formed hydrophobic domains. Therefore, a longer time is needed to overcome the hydrophobic interactions, leading to slower diffusion and sustained release.

**Figure 4:**
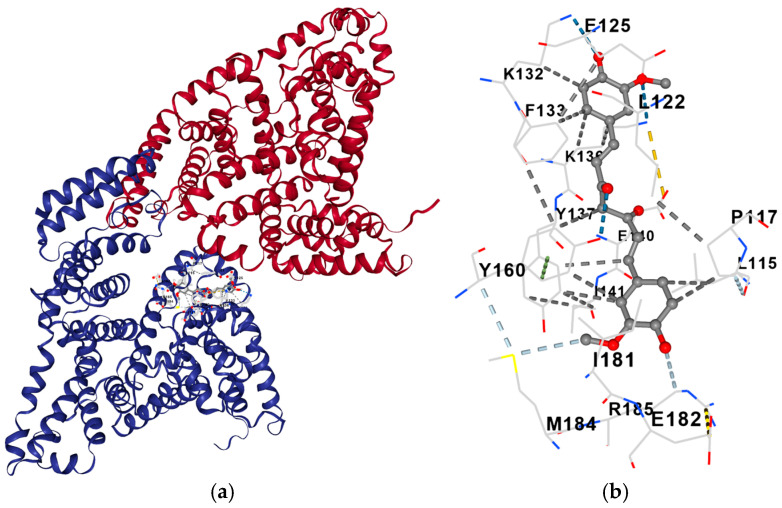
Three-dimensional presentation of the molecular docking (**a**) and presentation of the interactions between curcumin and albumin: hydrophobic interactions (grey colour) and hydrogen bonds (dark and light blue colour) (**b**).

In order to obtain insight into possible interactions between curcumin and albumin, molecular docking was conducted (Figure 4a). The binding energies and related cavity volumes are presented in Table 2. The results suggested prevalent hydrophobic interactions (grey colour) between curcumin and albumin (Figure 4b). Hydrogen (dark and light blue colour) bonds were also observed. The hydrophobic interactions occurred between the aromatic rings of curcumin and the nonpolar protein residues, while the hydrogen interactions are formed between its hydroxyl groups and the peptide backbone oxygens [34].

**Figure 5:**
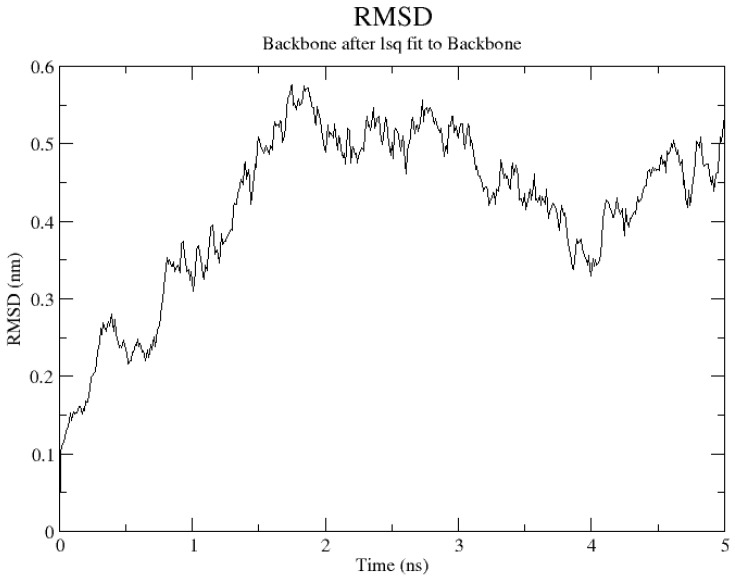
RMSD values (nm) vs. time (ns) during the simulation of curcumin–albumin complex.

**Figure 6:**
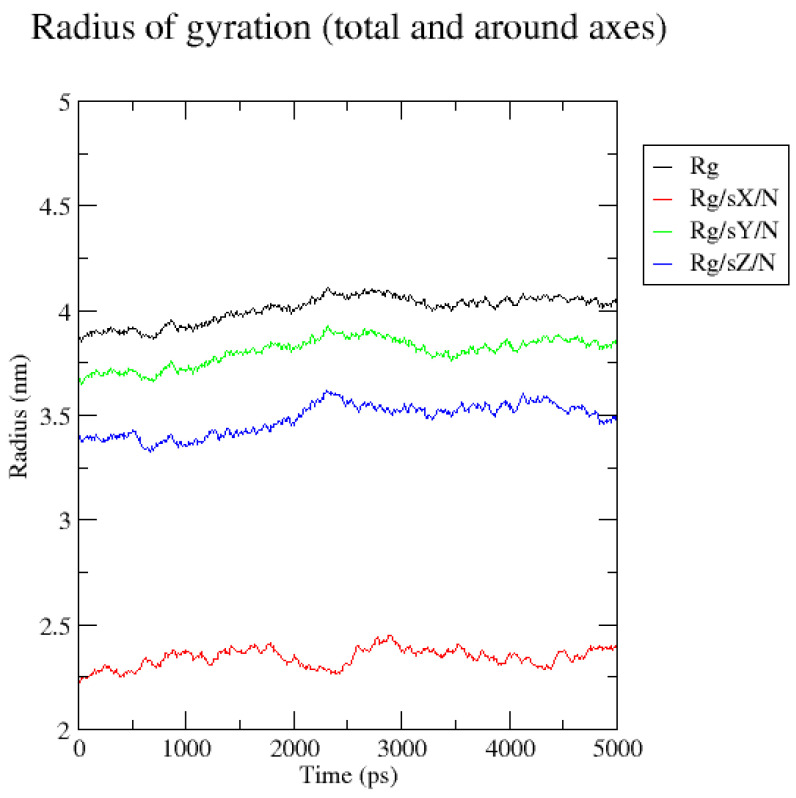
Radius of gyration (Rg) of the curcumin–albumin complex.

In order to further evaluate the binding between curcumin and albumin, a molecular dynamics simulation was conducted (raw data are presented as Supplementary Files S1–S3). As can be seen, after the initial rise, the RMSD (root mean square deviation) value stabilizes at around 0.3 to 0.5 nm (Figure 5). This showed that the protein has reached a relatively stable conformation since it was not significantly deviating from its original conformation. It is important to note that the larger proteins can exhibit slightly higher RMSD values, without indicating instability. Albumin could be considered a large protein, since the number of amino acid residues is more than 200 [35]. The radius of the gyration data extracted from the simulation test showed a stable overall value, since there were no large fluctuations (Figure 6). This suggested that the structure of the protein–ligand complex remained relatively constant during the simulation of 5 ns. In conclusion, the data suggested a relatively stable structure during the simulation.

### 2.3. Diffuse-Reflectance UV–Vis and XRD Analyses

**Figure 7:**
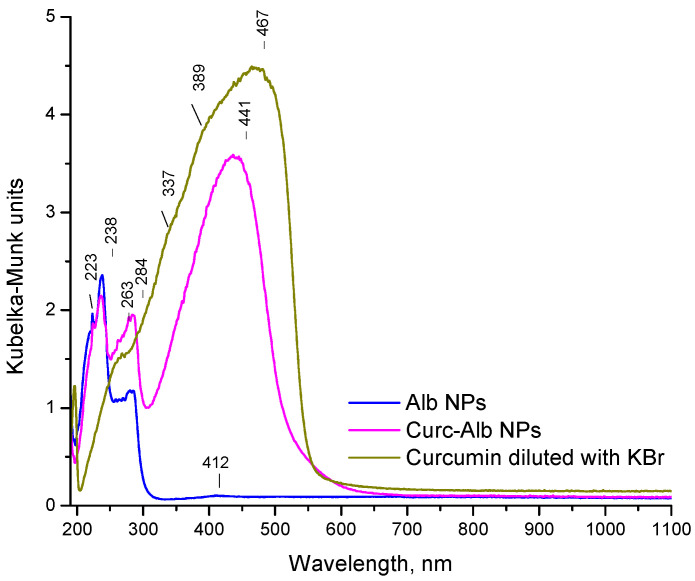
UV-Vis spectra of curcumin, pure albumin (Alb), empty albumin nanoparticles (AlbNPs), and loaded curcumin–albumin nanoparticles (CurcAlbNPs).

The successful loading of curcumin was confirmed by the UV−Vis analyses (Figure 7). The spectrum of pure curcumin showed several overlapped characteristic bands with maxima around 238 and 467 nm. The first one was attributed to the π→π* transition of the aromatic benzene rings of curcumin. The intensive band at 467 nm was due to the extended conjugation across the entire curcumin molecule (aromatic rings connected via a conjugated diketone bridge). This band lies in the visible region and gives curcumin its yellow-orange colour. The 223 nm and 238 nm bands in the albumin nanoparticles spectrum reflected the protein secondary structure and backbone conformations. The band at 284 nm could be due to the environment of aromatic residues, particularly tryptophan, and is sensitive to folding, denaturation, or ligand binding. The spectrum of the albumin–curcumin complex combined the features of the above-described spectra, indicating the formation of the complex between curcumin and albumin nanoparticles. The band shifts in the region of 238–284 nm suggested conformational changes in albumin upon curcumin binding. The red shift in the curcumin chromophore band to 441 nm suggested that curcumin was encapsulated or embedded in albumin nanoparticles via strong interaction (likely hydrophobic, van der Waals, and hydrogen bonding). This confirmed the results from the molecular docking.

**Figure 8:**
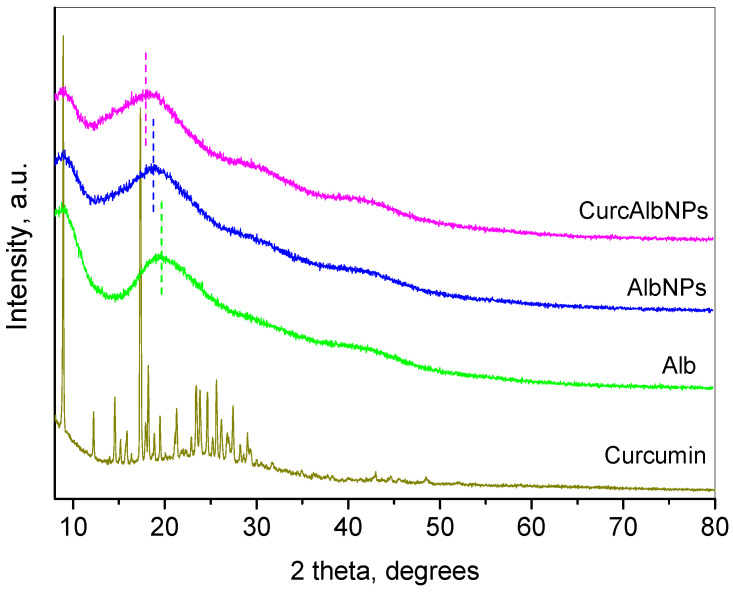
XRD pattern of curcumin, pure albumin (Alb), empty albumin nanoparticles (AlbNPs) and loaded curcumin–albumin nanoparticles (CurcAlbNPs).

Figure 8 presents the XRD pattern of curcumin, pure albumin, albumin nanoparticles and loaded curcumin on the albumin nanoparticles. The diffraction pattern of curcumin represented many sharp, intense peaks consistent with the ICDD-PDF # 9-816 reference pattern of this compound. The crystal structure was defined as a monoclinic SG P2/n with refined unit cell parameters a = 12.689(1) Å, b = 7.2163(5) Å, c = 19.837(2) Å and beta = 95.53(1) Å. The mean crystallite size was determined to be 109 ± 2 nm. The obtained values for the unit cell parameters were close to those given in the literature [36].

The X-ray diffraction pattern of the albumin sample was represented as three amorphous humps centred at 19.6, 30.3, and 42.2 degrees 2θ, respectively. The pattern of albumin nanoparticles (AlbNPs) repeated the main view of the previous pattern, but with a slight shift in the humps toward lower angles (18.8° 2θ) being registered. The XRD pattern of the complex (CurcAlbNPs) showed the amorphization of the curcumin phase combined with an additional shift in the maxima of the amorphous humps of the albumin (18.1° 2θ), indicative of the elongation of its internal bonds at the expense of the bonds with curcumin.

### 2.4. DPPH (2,2-Diphenyl-1-Picrylhydrazyl) and ABTS (2,2′-Azino-Bis(3-Ethylbenzothiazoline-6-Sulfonic Acid)) Tests

**Figure 9:**
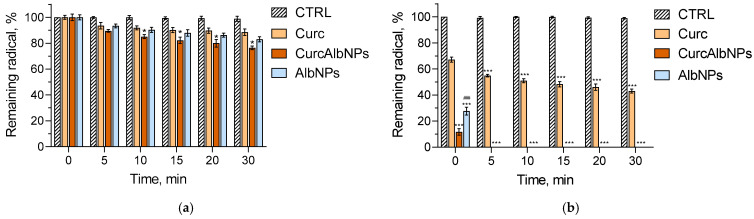
Radical-scavenging activity of free curcumin (Curc), encapsulated curcumin (CurcAlbNPs), and empty albumin nanoparticles (AlbNPs) against DPPH (**a**) and ABTS (**b**) radical. * *p* < 0.05 and *** *p* < 0.001 vs. Curc group; ### *p* < 0.001 vs. CurcAlbNPs group.

The radical-scavenging activity of the non-loaded drug (Curc), loaded in the nanoparticles curcumin (CurcAlbNPs) and empty nanoparticles (AlbNPs), was evaluated by performing the DPPH and ABTS assays. The results from both tests showed that the radical-scavenging activity of the encapsulated curcumin was stronger than that of the pure drug (Figure 9). Interestingly, the empty nanoparticles also showed high scavenging activity. It has been reported that bovine serum albumin showed significant activity against ABTS radical, mainly due to the presence of the amino acid tyrosine [37]. Regarding the DPPH, after 30 min of incubation, there was approximately 12%, 24%, and 17% scavenging activity for Curc, CurcAlbNPs, and AlbNPs, respectively (Figure 9a). This tendency was more pronounced against the ABTS radical, where the encapsulated curcumin and empty nanoparticles showed complete scavenging of the radical after 5 min of incubation (Figure 9b). For comparison, the non-encapsulated drug scavenged 53% of the radical for 30 min. These results are an indication of the opportunity to enhance the antioxidant effect of curcumin due to the antioxidant activity of the carrier. Possible explanation for the different effect against both radicals is the slower initial electron transfer in the DPPH assay, due to the sterically hindered DPPH radical site, making it difficult to access, as well as the higher reactivity and sensitivity of ABTS [38,39]. Therefore, the incorporation of curcumin in the albumin nanoparticles could be considered a notably appropriate strategy for enhancing its antioxidant potential in the treatment of skin disorders.

### 2.5. In Vitro Model of Oxidative Stress

**Figure 10:**
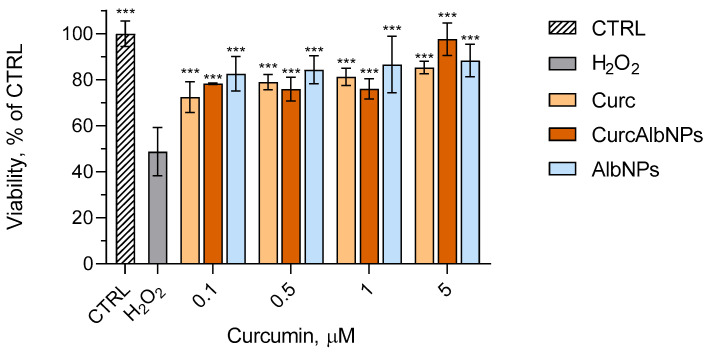
In vitro protective effects of non-loaded (Curc), encapsulated curcumin (CurcAlbNPs), and empty albumin nanoparticles (AlbNPs) against H_2_O_2_-induced oxidative stress in L929 fibroblasts. *** *p* < 0.001 vs. H_2_O_2_-treated group.

Taking into consideration the potential of the albumin nanoparticles to enhance the radical-scavenging activity of curcumin, the next step was to evaluate the protective effects of both the pure and encapsulated antioxidant in an in vitro model of oxidative stress in L929 mouse fibroblasts (Figure 10). The concentration range of curcumin was 0.1–5 µM, aiming to evaluate concentrations lower than its IC_50_ in L929 cells [40,41]. As can be observed, all samples showed antioxidant protective effect against the H_2_O_2_-induced cell damage. The highest cell viability was observed at 5 µM concentration with CurcAlbNPs showing a tendency for the most pronounced protective effect. Taking into consideration the slow release of curcumin from the albumin nanoparticles, the results showed that the encapsulation does not delay its antioxidant effects. This confirms that albumin has positive effects on curcumin’s antioxidant activity. Indeed, the empty nanoparticles also showed a protective activity in the cell model. This result correlated with previously reported data that albumin reduces oxidative stress in living cells through direct neutralization of free radicals such as hydroxyl, peroxyl, superoxide radicals, and singlet oxygen, as well as through the inhibition of NADPH oxidase [42].

### 2.6. Mechanical Properties of the Patches

**Figure 11:**
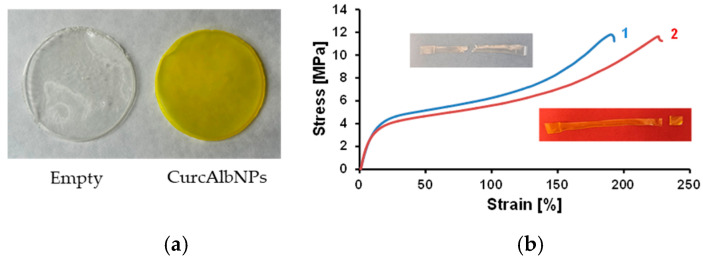
Digital images (**a**) of the developed empty (1) and nanoparticle-loaded patch (2) and stress–strain curves of both patches (**b**).

**Figure 12:**
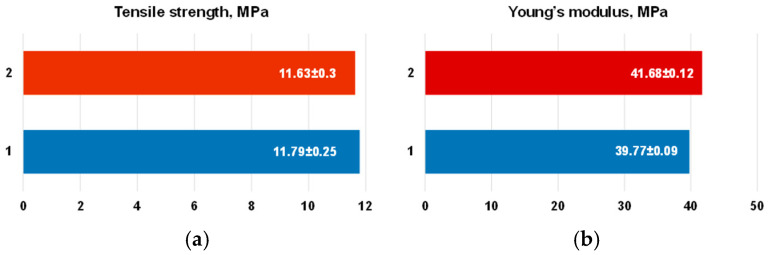
Mechanical properties of empty (1) and nanoparticles loaded film (2): tensile strength (**a**) and modulus of elasticity (**b**).

The CurcAlbNPs were then loaded into polyvinyl alcohol (PVA) patches for skin application (Figure 11a). It was reported that PVA is suitable for skin application and widely used for the preparation of films alone or in combination with other polymers since it is biocompatible, non-toxic, hydrophilic, and possesses sufficient mechanical strength [19,26]. Mechanical properties of thin films, such as tensile strength, elongation and elastic modulus, differ from their bulk counterparts due to size effects and microstructure. Determining and understanding these properties is crucial for the design of the patches used for medical applications. The mechanical properties of the patches obtained in the present study were determined using a universal testing system. The stress–strain curves of the empty patch (1) and the nanoparticles-loaded patch (2) are shown in Figure 11b. The measured values of the tensile strength of the two samples were very similar. The tensile strength of the tested empty patch was 11.79 ± 0.25 MPa, while the tensile strength of the nanoparticle-loaded patch was 11.63 ± 0.3 MPa (Figure 12a). The results obtained showed that patches 1 and 2 had similar values of tensile strength, revealing good mechanical properties, but patch 2 showed a higher value of elongation (226%) than patch 1 (190%). The addition of curcumin-containing albumin nanoparticles to patch 2 resulted in a slight decrease in the Young’s modulus (Figure 12b). The difference in elongation and Young’s modulus indicated that patch 2 was softer and more easily deformed under stress. Thus, it can withstand the same maximum load as patch 1, but its deformation before breaking is more pronounced. It should be taken into consideration that mechanical properties are crucial for the application of topical patches for wound care or the treatment of skin disorders. Mechanical requirements for topical patches include sufficient strength and flexibility (tensile strength, Young’s modulus, elongation at break) to withstand handling, a suitable thickness to ensure patient comfort and performance, and good adhesion to the skin. There are no universal standards for topical patch tensile strength; however, studies suggest a desirable range should match or exceed those of the human skin. For topical patches, the desired mechanical requirements include a tensile strength between 1 and 32 MPa [43] and a Young’s modulus from approximately 0.05 MPa to 60 MPa [44]. The mechanical properties of the hydrogel patch prepared in the present study are within the cited ranges. Thus, the patch could be considered appropriate for skin applications.

### 2.7. In Vitro Release Study of Curc and CurcAlbNPs Loaded in the Patch

**Figure 13:**
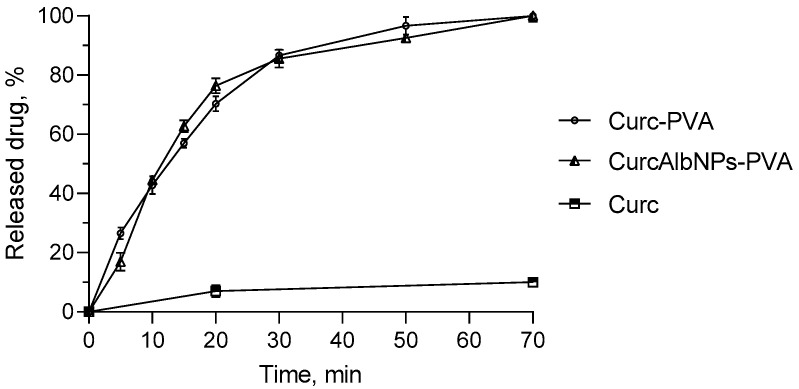
In vitro dissolution profiles in medium with pH = 5 of pure curcumin (Curc), curcumin formulated in the PVA patch (Curc-PVA), and encapsulated curcumin in the PVA patch (CurcAlbNPs-PVA).

The patches were evaluated regarding the release of non-encapsulated or encapsulated curcumin. Both patches were characterized by an equal release rate, in particular, approximately 85% after 30 min and a full release after 70 min (Figure 13). Thus, the drug release from the patches was faster than the release from the nanoparticles. In our opinion, the faster release was due to the surface-active properties of PVA and the related decrease in surface tension. Similar release rates from PVA patches were also observed for *Vitis vinifera* extract (30 min) [45], salicylic acid (60 min) [46], and Coenzyme Q10 (35 min) [47]. On the other hand, the difference between the release profiles from the albumin nanoparticles and that from the patch could be explained by the different methods applied for the release tests. Further, comparing the release of pure curcumin with that from the patch showed that, for 70 min, only 10% of curcumin in suspension was dissolved in the medium. These results indicated that the patch containing the drug-loaded nanoparticles is appropriate for skin application since it provided complete release of curcumin. In addition, the mechanical strength, flexibility, and adhesion properties of PVA would provide optimal residence on the skin.

## 3. Conclusions

In the present study, curcumin-loaded albumin nanoparticles were successfully prepared (encapsulation efficiency of 88.2%) via the simple desolvation method because of hydrophobic interactions between curcumin and albumin. The encapsulation of curcumin in the nanoparticles resulted in a higher in vitro radical scavenging activity against the ABTS radical compared to that of the pure drug. Similarly, the antioxidant potential of the encapsulated curcumin in the model of H_2_O_2_-induced oxidative stress in fibroblasts was trending stronger than the effect of pure curcumin. The nanoparticles were characterized by a small diameter (163 nm) and narrow size distribution that enables their further formulation in a PVA patch, which showed appropriate mechanical and biopharmaceutical properties for skin application.

## 4. Materials and Methods

### 4.1. Materials

Bovine serum albumin (fraction V), Curcumin (analytical standard grade, ≥98%), Dulbecco’s Modified Eagle’s Medium, fetal bovine serum (FBS), L-glutamine, 3-(4,5-dimethylthiazol-2-yl)-2,5-diphenyltetrazolium bromide (MTT), 2,2-diphenyl-1-picrylhydrazyl (DPPH), 2,2′-azino-bis(3-ethylbenzothiazoline-6-sulfonic acid) (ABTS), and glycerin were purchased from Sigma-Aldrich (Merck KGaA, Darmstadt, Germany). Polyvinyl alcohol (PVA 22000) was provided by Fluka Chemie AG (Seelze, Germany). Dialysis membrane (standard-grade regenerated cellulose, 4–8 kDa MWCO, Spectrum Labs) was provided by Fisher Scientific (Göteborg, Sweden). The murine L929 fibroblast cell was obtained from the European Collection of Cell Cultures (ECACC, Salisbury, UK).

### 4.2. Preparation of Empty (AlbNPs) and Curcumin-Loaded (CurcAlbNPs) Albumin Nanoparticles

The albumin nanoparticles were prepared via the desolvation method with some modifications [48,49,50]. Bovine serum albumin (100 mg) was dissolved in 2 mL distilled water. Thereafter, 2.4 mL of acetone was dripped during stirring of the albumin solution. The mixture was stirred until complete evaporation of the acetone. For the loaded nanoparticles, different amounts of curcumin were dissolved in the acetone. The dispersion was then filtered (0.45 µM Nylon filter, Sigma-Aldrich, Merck KGaA, Darmstadt, Germany), and the filter was rinsed with 50% ethanol. The concentration of the non-loaded curcumin in the filtrate was evaluated via the HPLC method (Thermo Scientific UltiMate Dionex 3000, Chromeleon 7.2 SR3 Systems, Thermo Fisher Scientific, Waltham, MA, USA) [51]. The following conditions were applied: Aquasil C18 Column (250 mm × 4.60 mm, particle size 5 μm, pore size 100 Å), acetonitrile and 2% *v*/*v* acetic acid (50%:50% *v*/*v*) mobile phase, isocratic elution, injection volume of 20 µL, flow rate of 1 mL/min, retention time 17.9 min, a column temperature of 33 °C, and ultraviolet detection at 425 nm. The concentrations were calculated by applying a standard curve obtained in the range of 3–50 µg/mL (r > 0.9950). The following equations were used for the determination of encapsulation efficiency (EE) and loading degree (LD) of curcumin:

EE = (A − B)/A × 100,(1)

LD = (A − B)/C,(2)

where A is the total mass of curcumin, B is the mass of non-encapsulated curcumin, and C is the volume of curcumin-loaded nanodispersion.

### 4.3. Characterization of the Nanoparticles

The dynamic light scattering (DLS) method, utilizing a Zetasizer NanoBrook 90Plus PALS (Brookhaven Instruments Corporation, Holtsville, NY, USA) equipped with a 35 mW red diode laser (λ = 640 nm) at a scattering angle of 90°, was applied for evaluating the mean size and polydispersity of the nanoparticles. The zeta potential was determined by the phase analysis light scattering (PALS) method at a scattering angle of 15°.

Transmission electron microscopy (TEM, HR STEM JEOL JEM 2100, Tokyo, Japan) was used for confirmation of the size and shape of the particles.

X-ray patterns were collected on a Bruker D8 Advance diffractometer (Bruker AXS, Karlsruhe, Germany) with Cu Kα radiation and a LynxEye detector between 5 and 80 °2θ with a constant step of 0.02 °2θ. The identification of crystalline phases was performed with EVA software (version 5.2) using the ICDD database PDF2(2024). The mean crystallite size of curcumin was evaluated using peak broadening values obtained by the full profile fitting method implemented in the Topas 4.2 programme.

The diffuse-reflectance UV–vis spectra were obtained with a Thermo Scientific Evolution 300 spectrophotometer (Thermo Scientific GmbH, Dreieich, Germany) equipped with a Praying Mantis accessory.

### 4.4. In Vitro Release Studies

In vitro release studies of CurcAlbNPs and PVA patches containing pure and encapsulated curcumin were conducted by applying the dialysis method (4–8 kDa MWCO) in a shaking water bath (IKA Labortechnik HS-B20, Staufen, Germany) or Paddle method in a dissolution apparatus (Erweka DT 600, Heusenstamm, Germany), respectively. The tests were performed in a buffer with pH = 5.0 (containing 10% ethanol) at a temperature of 32 °C. At predetermined time intervals, samples (2 mL) were taken from the acceptor phase, and the concentration was evaluated via HPLC (Thermo Scientific UltiMate Dionex 3000 SD, Chromeleon 7.2 SR3 Systems, Thermo Fisher Scientific, Waltham, MA, USA) as described above. Equal amounts of fresh buffer were returned, aiming to maintain sink conditions.

### 4.5. Molecular Docking and Dynamics

CB-Dock2 [52] (https://cadd.labshare.cn/cb-dock2/index.php, accessed on 29 May 2025) which is based on AutoDock Vina (version 1.2.0) docking [53,54,55,56] was used for applying the protein–ligand docking method in order to evaluate the interactions between curcumin and albumin. Curcumin (CID 968516, PubChem) was used as a ligand and bovine serum albumin (4F5S, Protein Data Bank (PDB)) was used as a receptor. Information about the potential binding cavities and the related binding energies, as well as the type of interactions, is presented. The platform Visual Dynamics, which is a WEB application for molecular dynamics simulation using GROMACS, was applied to evaluate the stability of the formulated curcumin–albumin complex [57]. The following parameters were selected: Force field–AMBER99SB; Water model–SPC simple point charge; Box type–Cubic. The curcumin–albumin complex (.pdb) was obtained from CB-Dock2. The molecule of the complex was then separated (to ligand and receptor in the specific conformation) using UCSF ChimeraX software (version 1.11rc202511220640). Then, hydrogen atoms were added to the molecule of curcumin. The ACPYPE Server was applied to obtain the files of the ligand required for the molecular dynamics.

### 4.6. DPPH and ABTS Studies

The radical-scavenging activity of Curc, CurcAlbNPs, and AlbNPs was evaluated by applying the DPPH [58] and ABTS [59,60] assays. DPPH was dissolved in ethanol (440 µg/mL), while ABTS was mixed with potassium persulfate in an aqueous solution, incubated under dark conditions (25 °C for 16 h), aiming to activate the radical, and then diluted to 5% in ethanol. After that, 1 mL of the samples (42 µM curcumin and albumin in corresponding concentration to the dilution of the particles) was mixed with 1 mL DPPH or ABTS. Distilled water was used as a control sample. The absorbance was measured at 517 nm for the DPPH and at 734 nm for the ABTS radical (Thermo Fisher Scientific, Waltham, MA, USA). The results are normalized according to the control group, namely the radical mixed with water.

### 4.7. In Vitro Model of Oxidative Stress

The in vitro cell experiments were conducted on murine L929 fibroblasts. The cell line was maintained in Dulbecco’s Modified Eagle’s Medium (DMEM, low glucose), supplemented with 10% fetal bovine serum and 4 mM L-glutamine. Incubation under standard conditions (5% CO_2_, 37 °C, high humidity, Esco CelCulture^®^ CO_2_ Incubator, CCL-170B-8-IVF, Esco Micro Pte. Ltd., Singapore) was applied. The cells were subcultured according to the protocol for adherent cell lines [61], and passage 5 was used for the following experiments.

A hydrogen peroxide (H_2_O_2_)-induced model of oxidative stress on the murine L929 fibroblast cell line was conducted in order to evaluate the protective ability of pure curcumin (Curc), encapsulated curcumin (CurcAlbNPs), and empty nanoparticles (AlbNPs). The cells were seeded in 96-well plates at a cell density of 2 × 10^4^ and incubated overnight at 37 °C, 5% CO_2_, and high humidity (Esco CelCulture^®^ CO_2_ Incubator, CCL-170B-8-IVF, Esco Micro Pte. Ltd., Singapore). After 24 h of incubation, the L929 cells were treated with Curc (ethanol solution), CurcAlbNPs (0.1, 0.5, 1, and 5 µM concentrations of curcumin), or AlbNPs at corresponding concentrations for 2 h. Then, 500 µM H_2_O_2_ was applied for 1h in order to produce oxidative stress-related cell damage. PBS containing Ca^2+^ and Mg^2+^ was used for rinsing the cells, and then fresh medium was added. The cells were incubated again for 24 h. After that, a solution of 10 mg/mL MTT in PBS was added to each well and incubation at 37 °C for 3 h was performed. Thereafter, the MTT solution was aspirated, and 100 µL/well DMSO was added for dissolving the formed formazan crystals. The absorbance was measured at 570 nm (690 nm for background absorbance) using a multiplate reader, Synergy 2 (BioTek Instruments, Inc., Highland Park, Winooski, VT, USA).

### 4.8. Preparation of PVA Patches Containing Curcumin-Loaded Nanoparticles

The casting method was applied for the preparation of 10% PVA patches [62,63]. Briefly, PVA was dissolved in the required amount of distilled water at 90–95 °C under gentle stirring. Then glycerol (3% wt/wt) was added as a plasticizer. The mixture was gently stirred, aiming to avoid air inclusion, poured into a Petri dish (diameter = 9.8 cm), and left to dry (4 h at 80 °C). For the patches containing curcumin, the aqueous dispersion of the nanoparticles or alcoholic solution of pure curcumin (15% wt/wt) were added to the required amount of water.

### 4.9. Characterization of the Mechanical Properties of PVA Patches

A single-column INSTRON 3344 universal testing system (Illinois Tool Works Inc., Glenview, IL, USA) with a 50 N load cell and Bluehill Universal software was used to evaluate the mechanical properties of the patches. The strain rate was 10 mm/min, the starting distance between clamps was 40 mm, and the ambient temperature was 21 °C. The patches’ width and length were 8 mm and 60 mm, respectively. A digital thickness gauge FD-50 (Käfer Messuhrenfabrik GmbH & Co., KG, Villingen-Schwenningen, Germany) was used to determine the thickness of each sample before the tensile test. The measured sample thickness was ~400 μm. The average values of tensile strength (MPa), elongation at break (%), and Young’s modulus (MPa) were determined from ten specimens of each patch that were assessed for statistical significance.

### 4.10. Statistical Analyses

In the present study, the experiments were conducted in triplicate. The results are expressed as mean values ± SD. Statistical analyses were performed using the GraphPad Prism 8 Software (Dotmatics, San Diego, CA, USA). The ROUT method was applied for identifying the outliers. One-way ANOVA with Tukey’s or Dunnett’s multiple comparison post-test was conducted for the analysis of the data regarding the encapsulation efficiency and loading degree, or for the comparison with the H_2_O_2_-treated group in the in vitro cell experiment, respectively. Multiple *t*-tests with Holm–Sidak correction were applied for comparing the different groups in the ABTS and DPPH assays as well as in the in vitro model of oxidative stress. *p* < 0.05 was considered significant.

## Figures and Tables

**Table 1:** Mean size, polydispersity index (PDI), and zeta potential of empty and curcumin-loaded albumin nanoparticles.

Nanoparticles	Mean Size, nm	PDI	Zeta Potential, mV
Empty	117 ± 5.5	0.286	−27.5
Loaded	163 ± 6.5	0.234	−28.5

**Table 2:** Binding energy and related cavity size for curcumin and albumin.

Vina Score	Cavity Volume (Å^3^)	Centre (x, y, z)	Docking Size (x, y, z)
−8.8	4101	49, 22, 93	35, 26, 26
−8.2	3494	20, 24, 101	26, 26, 35
−8.1	8957	68, 22, 85	35, 35, 26
−7.9	8389	0, 26, 108	35, 35, 26
−6.6	18,855	47, 22, 119	35, 35, 26

## Data Availability

The original contributions presented in this study are included in the article/Supplementary Material. Further inquiries can be directed to the corresponding authors.

## Supplementary Materials

The following supporting information can be downloaded at: https://www.mdpi.com/article/10.3390/gels11120979/s1. File S1: GROMACS Commands; File S2: RMSD raw data; File S3: Radius of gyration raw data.
